# Biomechanical Characterisation of Bone-anchored Implant Systems for Amputation Limb Prostheses: A Systematic Review

**DOI:** 10.1007/s10439-017-1976-4

**Published:** 2018-01-11

**Authors:** Alexander Thesleff, Rickard Brånemark, Bo Håkansson, Max Ortiz-Catalan

**Affiliations:** 1 Biomechatronics and Neurorehabilitation Laboratory, Department of Electrical Engineering, Chalmers University of Technology, Gothenburg, Sweden; 2 Integrum AB, Mölndal, Sweden; 3 International Centre for Osseointegration Research, Education and Surgery (iCORES), Department of Orthopaedics, University of California, San Francisco, CA USA; 4 Department of Orthopaedics, Gothenburg University, Gothenburg, Sweden

**Keywords:** Bone-anchored prostheses, Osseointegration, Direct skeletal attachment

## Abstract

Bone-anchored limb prostheses allow for the direct transfer of external loads from the prosthesis to the skeleton, eliminating the need for a socket and the associated problems of poor fit, discomfort, and limited range of movement. A percutaneous implant system for direct skeletal attachment of an external limb must provide a long-term, mechanically stable interface to the bone, along with an infection barrier to the external environment. In addition, the mechanical integrity of the implant system and bone must be preserved despite constant stresses induced by the limb prosthesis. Three different percutaneous implant systems for direct skeletal attachment of external limb prostheses are currently clinically available and a few others are under investigation in human subjects. These systems employ different strategies and have undergone design changes with a view to fulfilling the aforementioned requirements. This review summarises such strategies and design changes, providing an overview of the biomechanical characteristics of current percutaneous implant systems for direct skeletal attachment of amputation limb prostheses.

## Introduction

Conventionally, a limb prosthesis is attached to the stump of an amputee by the use of a socket, which suspends the prosthesis to the stump by compressing over soft tissues. However, the socket-stump interface often causes such complications as poor fit, discomfort, skin problems, sweating, and pressure sores.^19^,^29^,^52^,^58^,^70^ An alternative way to attach limb prostheses to the human body is to bypass the soft-tissue in the stump for direct load transfer to the skeletal system. In this concept, a percutaneous implant system is surgically implanted with its proximal end directly into bone tissue in the stump. The distal end of the implant system extends percutaneously from the residual limb and allows for the attachment of an external prosthesis. This eliminates the need for a compression socket and eliminates well-known socket-associated problems. Additional benefits of direct skeletal attachment of limb prostheses reported in the literature include improved range of motion,^31^,^74^ walking ability,^24^,^32^ sitting comfort,^31^ reduced energy expenditure,^76^ and improved awareness* via* osseoperception.^33^,^42^ In the 1960s and 1970s, several attempts were made on animals to achieve direct skeletal attachment of limb prostheses. In most of these experiments, intramedullary rods of stainless steel or cobalt-chrome-molybdenum alloy were used,^35^,^36^ however, no long-term successful results were reported. Sandblasted surface treatment, porous ceramic coatings,^35^,^36^ composite materials of glass fibres or carbon fibres in plastic matrices,^36^ and alternative designs with supra-cortical-, or supra-periosteal attachment,^36^–^38^ to the bone have also been tried, without satisfactory outcomes. In 1977, Mooney reported on unsuccessful attempts on three human transhumeral amputees with intramedullary stainless steel implants fixed with bone cement.^59^ Most of the failures have been attributed to infection at the skin penetration site and loosening at the bone-implant interface.

In the early 1960s, P.-I. Brånemark discovered the ability of bone tissue to closely adhere and form a strong mechanical bond to titanium. He introduced the term osseointegration as direct anchorage to bone tissue to describe this close contact.^16^ Brånemark pioneered the use of titanium as implant material for dental prostheses in the treatment of edentulous patients, with positive clinical results.^16^ From these findings, the concept was later extended to the orthopaedic field when an implant system for direct skeletal attachment of limb prostheses was developed and implanted in a bilateral transfemoral amputee in Sweden for the first time in 1990.^21^ The system was further developed to accommodate other amputation levels. Early implants were custom designed until 1999, when the Swedish system was introduced to the market under the name OPRA (Osseointegrated Prostheses for the Rehabilitation of Amputees, Integrum AB, Mölndal, Sweden). The OPRA implant system is currently available in 12 countries. Following the successful results in Sweden, another implant system was independently developed in Germany under the name of ILP (Integral Leg Prosthesis, Orthodynamics GMbH, Lübeck, Germany). In 1999, the first patient was treated with the ILP implant system,^10^ which is now in clinical use in Germany, the Netherlands, and Australia. Another system, based on the ILP design, was recently developed in Australia under the name of OPL (Osseointegrated Prosthetic Limb, Permedica s.p.a., Milan, Italy). This system is also clinically available in The Netherlands. To date OPRA, ILP, and OPL are the only commercially available systems for direct skeletal attachment of external limb prostheses. However, a number of newer systems are under development, four of which have reached the stage of clinical experiments in humans. These are the ITAP^71^ (Intraosseous Transcutaneous Amputation Prosthesis, Stanmore Implants Worldwide, Watford, United Kingdom) developed in the United Kingdom, the Keep Walking Advanced^27^ (Tequir S.L., Valencia, Spain) developed in Spain, and two systems developed in the United States: POP^20^ (Percutaneous Osseointegrated Prosthesis, DJO Global, Austin, USA) and COMPRESS^57^ (Zimmer Biomet, Warsaw, USA). In addition to these systems, implantation of a custom-made implant for attachment of an external prostheses in a transfemoral amputee has been reported in a single case study in the USA.^39^ A number of other systems have been tested in animal studies, but only the AEAHBM (Alameda East Animal Hospital BioMedtrix, Boonton, USA) developed in the USA, has shown successful outcome for a load-bearing prosthetic limb for more than one year.^18^

Previous reviews have focused on clinical^78^ and functional outcomes,^54^ as well as the design features of the implant systems.^67^ The present review aims to characterise the biomechanical interfaces between the implant and biological tissue, and between individual components within each implant system. In this article, the term ‘implant system’ refers to implanted and percutaneous components, including external safety devices, to which an external prosthesis can be attached.

## Methods

**Figure 1:**
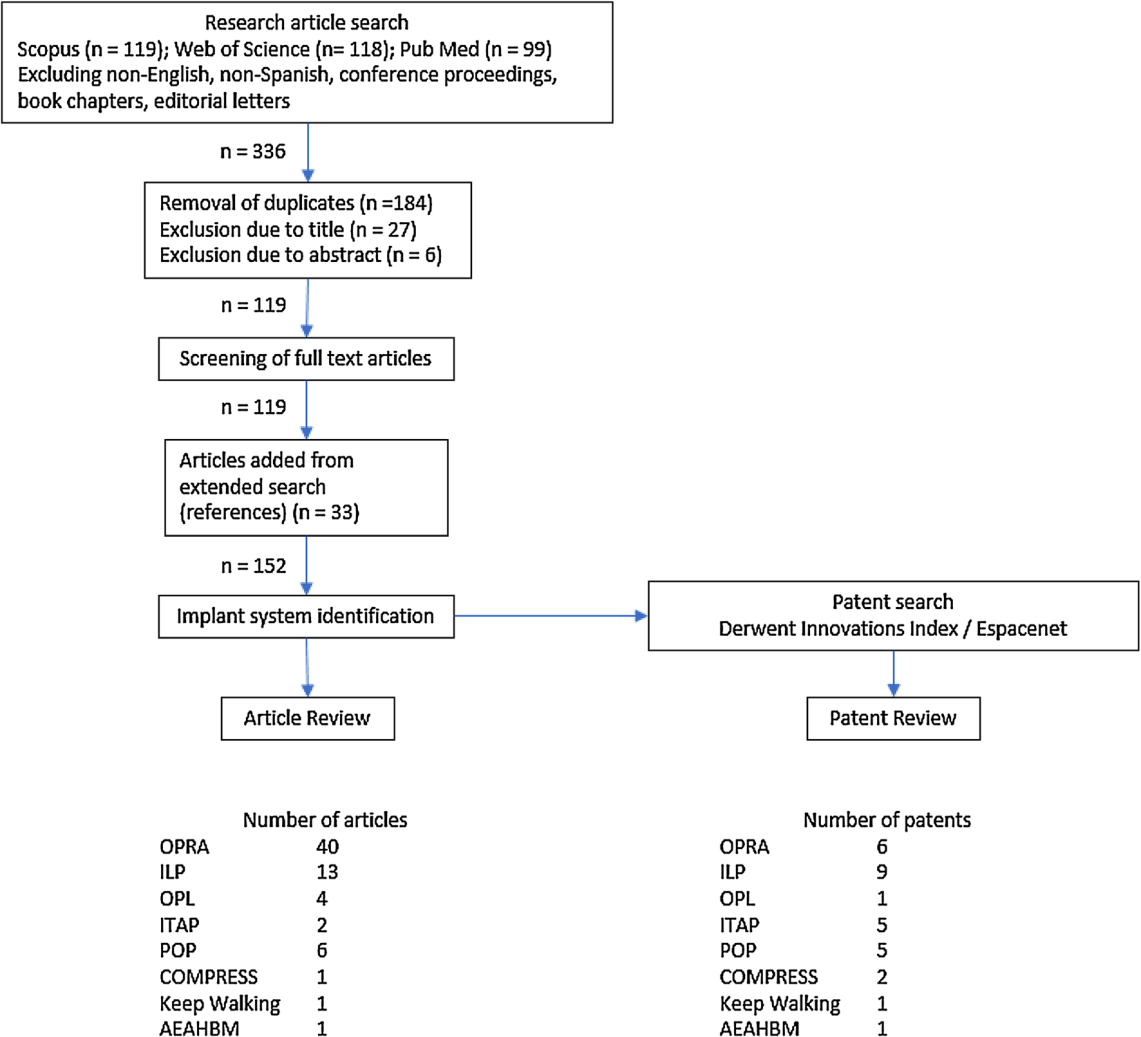
Schematic view of the methodology used for the literature review and the search results. From the filtered article search, the number of articles per implant system was determined according to the following criterion. In order to be counted as an article for a particular implant system, the article had to describe the mechanical properties of the implant system or report results from implantation of the implant system for direct skeletal attachment of a load-bearing artificial limb.

A systematic literature review was performed using the three databases: *Scopus*, *Web of Science*, and *PubMed*. Article title, key words, and abstracts were searched using the following search condition: (osseointegrat* OR “skeletal attachment” OR bone?anchored) AND (limb OR prosthes*) AND (amput*) AND (implant*). The inclusion criteria for the articles required them to contain a description of an implant system that allows for direct skeletal attachment of a load-bearing artificial limb. Load-bearing limbs were defined as upper or lower legs and arms. Journal articles published prior to 1 April 2017 were considered. Conference proceedings, book chapters, editorial letters, non-English, and non-Spanish articles were excluded. The screening procedure is presented in detail in Fig. 1. The filtered search yielded 152 unique and relevant articles in total. The criteria for inclusion was defined as implant systems that (1) allow for direct skeletal attachment of a load-bearing artificial limb and (2) have been evaluated in human or animal models of a load-bearing artificial limb with successful function for at least 1 year. We identified eight relevant implant systems that fulfilled these criteria. In addition, a patent search in *Derwent Innovations Index* and *Espacenet* was performed to obtain further information about these implant systems. Only patents describing the components of the previously identified implant systems were considered. The peer-reviewed research articles and patents were studied to determine the characteristics of each of the implant systems presented here.

## Results

**Figure 2:**
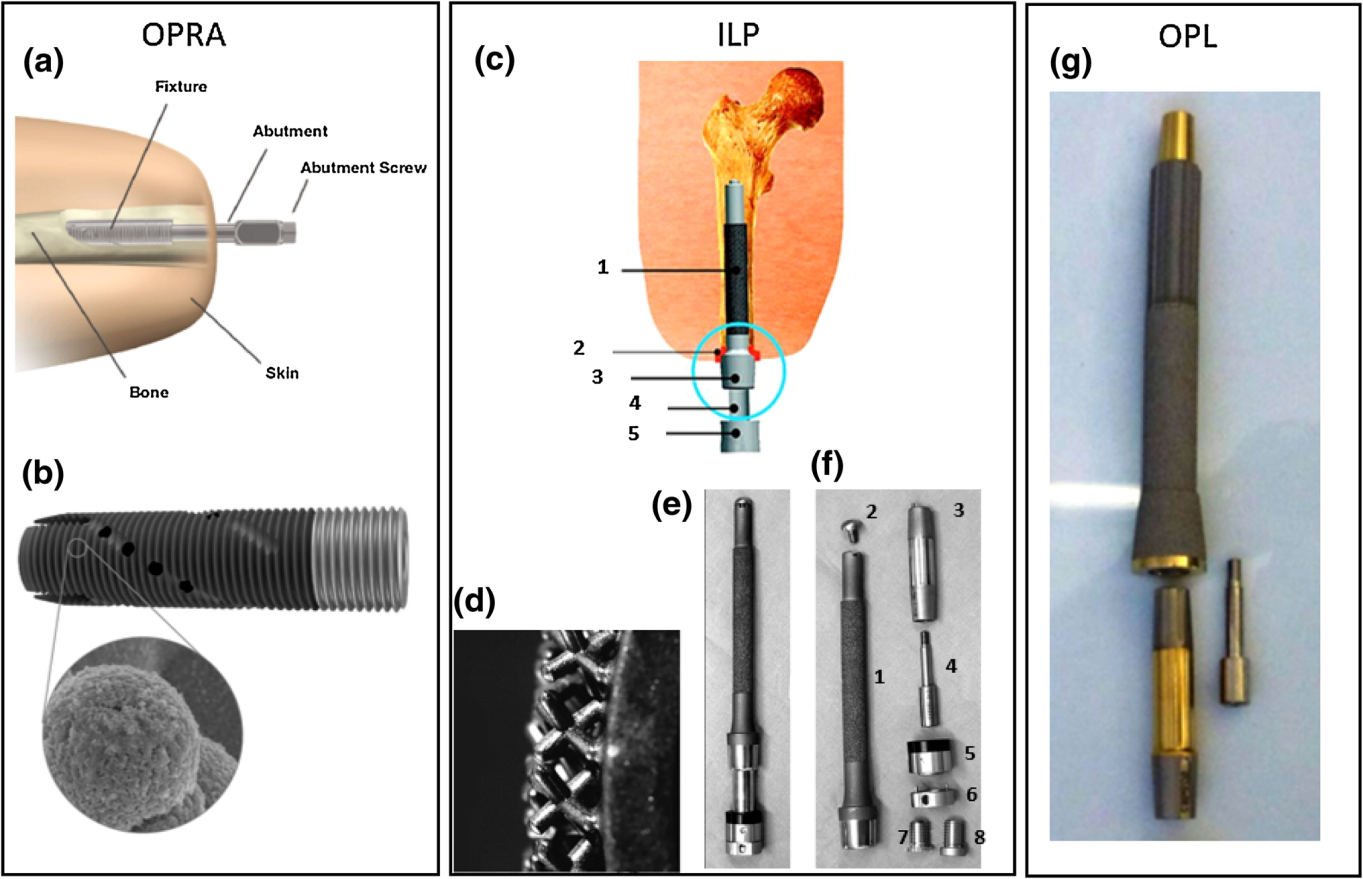
(a) Schematic image of OPRA implant system in an amputated limb; (b) OPRA Fixture; the exterior surface in the dark grey region is treated to enhance osseointegration. The lower image shows a close-up of the laser-induced micro structure from the surface treatment; (c) Schematic image of the ILP implant system: (1) Porous coated portion of the intramedullary component of the implant system, (2) inner lining, (3) Morse taper, (4) dual cone adapter, (5) knee connecting adapter. The red line indicates the stoma channel; (d) Close-up of the spongiosa metal surface to enhance osseointegration and ingrowth; (e) ILP implant system assembled; (f) Exploded view of ILP implant system assembly consisting of: (1) intramedullary implant, (2) temporary cover screw, (3) dual cone adapter, (4) safety screw, (5) sleeve, (6) rotating disc (until prosthetist has made final adjustments), (7) final propeller screw, (8) provisional screw; (g) OPL type-B implant system. Images A and B are published with courtesy of Integrum AB. Images C, D, E and F are reprinted from Journal of Rehabilitation Research & Development.^49^ Image G is reprinted from Unfallchirurg^6^ with permission from Springer.

**Figure 3:**
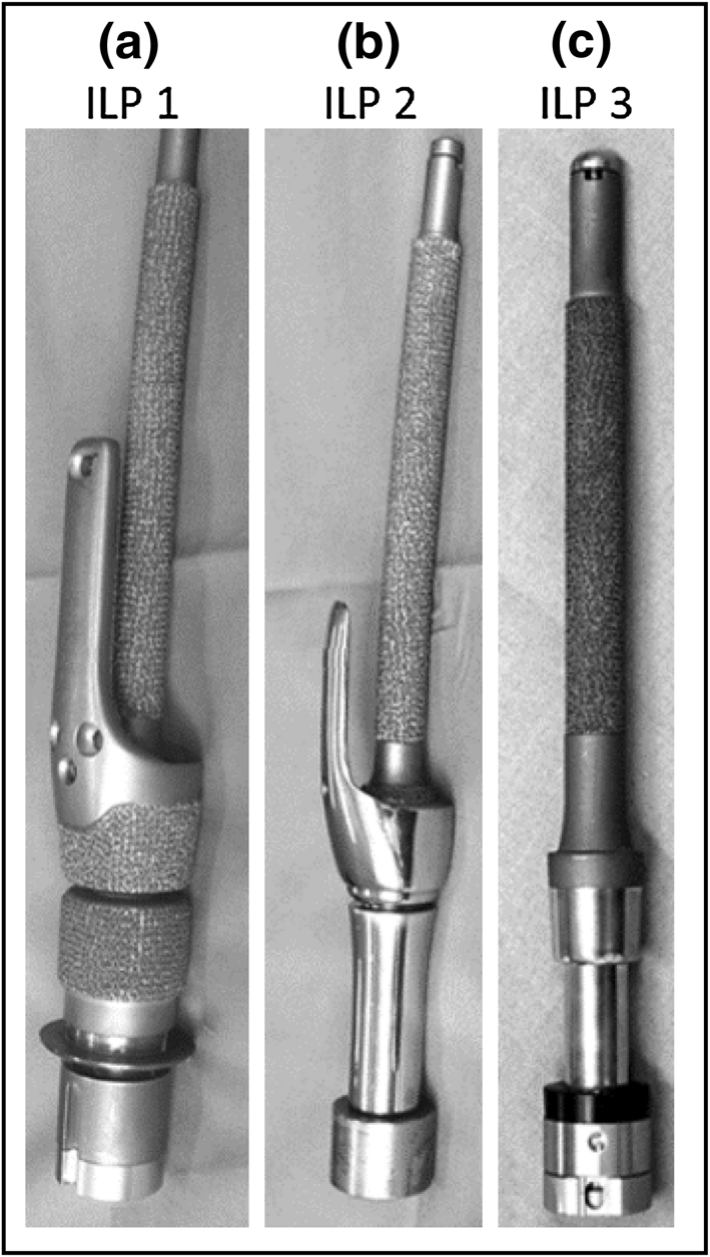
Design changes over time for the ILP implant. (a) First ILP implant design. The material is medical-grade cobalt-chrome alloy. The implant has a rough surface on both the intramedullary region and the subdermal region. A bone-stabilising bracket for improved fatigue properties; (b) Second implant design. Rough surface on distal post and bracket removed. Bracket, distal portion of implant and connector reduced in size; (c) Third implant design. Bracket removed. Revised connection to implant to a dual cone connection. Reprinted from Journal of Rehabilitation Research & Development.^49^

**Figure 4:**
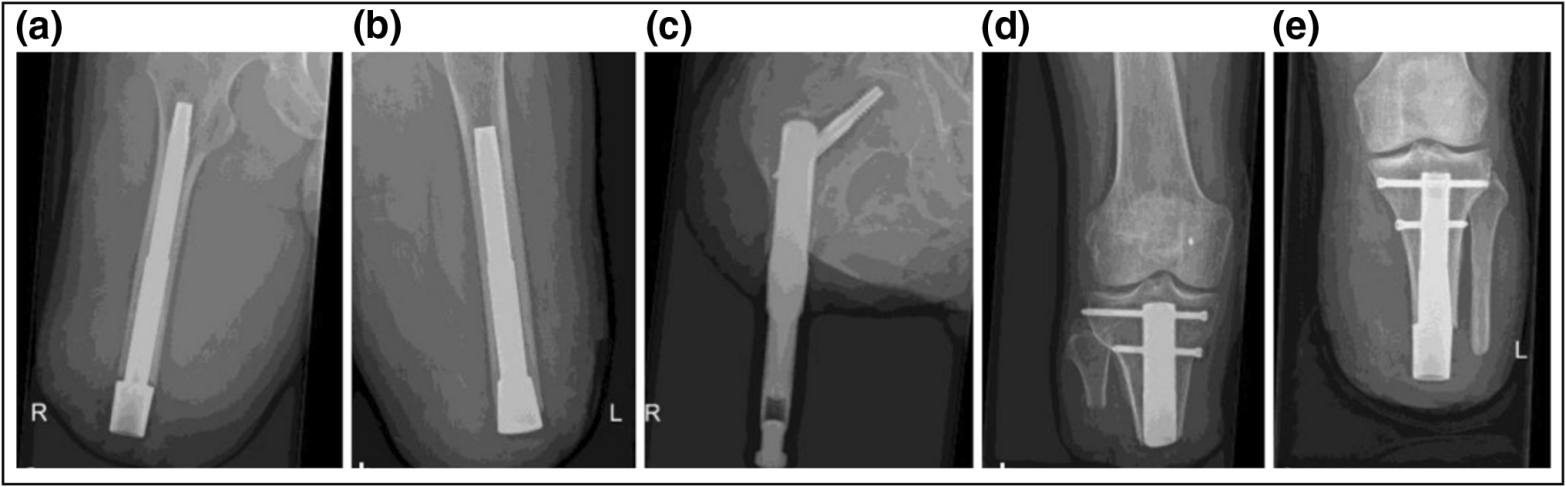
Examples of available OPL implants. (a) OPL type A with distal niobium polished extramedullary head; (b) OPL type B with an intramedullary distal head; (c–e) Custom-made implants with macroporous 3D mesh coating for accelerated osseointegration. Reprinted from Unfallchirurg.^23^

**Figure 5:**
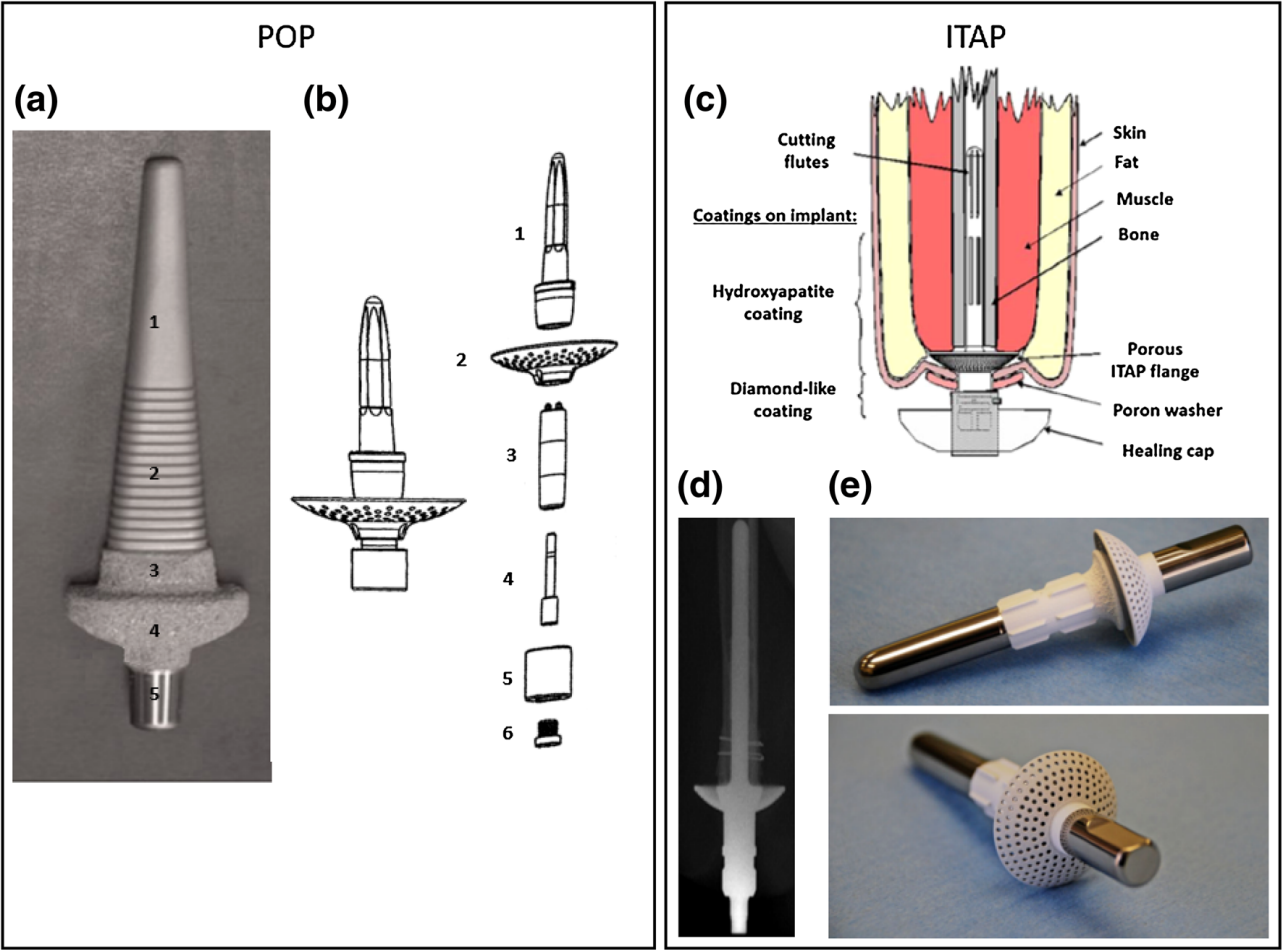
(a) Single-component POP implant used in sheep studies: (1) tapered smooth region, (2) fluted region, (3) porous coated region, (4) porous coated subdermal barrier, (5) Morse taper for connection to exo-prosthesis; (b) POP implant system assembly for humans: (1) implant stem, (2) stoma shield, (3) percutaneous post, (4) assembly bolt, (5) adapter, (6) adapter bolt; (c) Schematic view of the ITAP implant; (d) Radiograph of ITAP implant in a transhumeral amputee; (e) ITAP implant used in dog number 3 of the clinical study on dogs. Image A is reprinted from Clinical Orthopaedics and Related Research,^47^ with permission from Springer. Image B is reprinted from US Patent 9,433,505.^12^ Images C and D are reprinted from Journal of Hand Surgery^50^ with permission from Elsevier. Image E is reprinted from Veterinary Surgery^22^ with permission from John Wiley and Sons.

**Figure 6:**
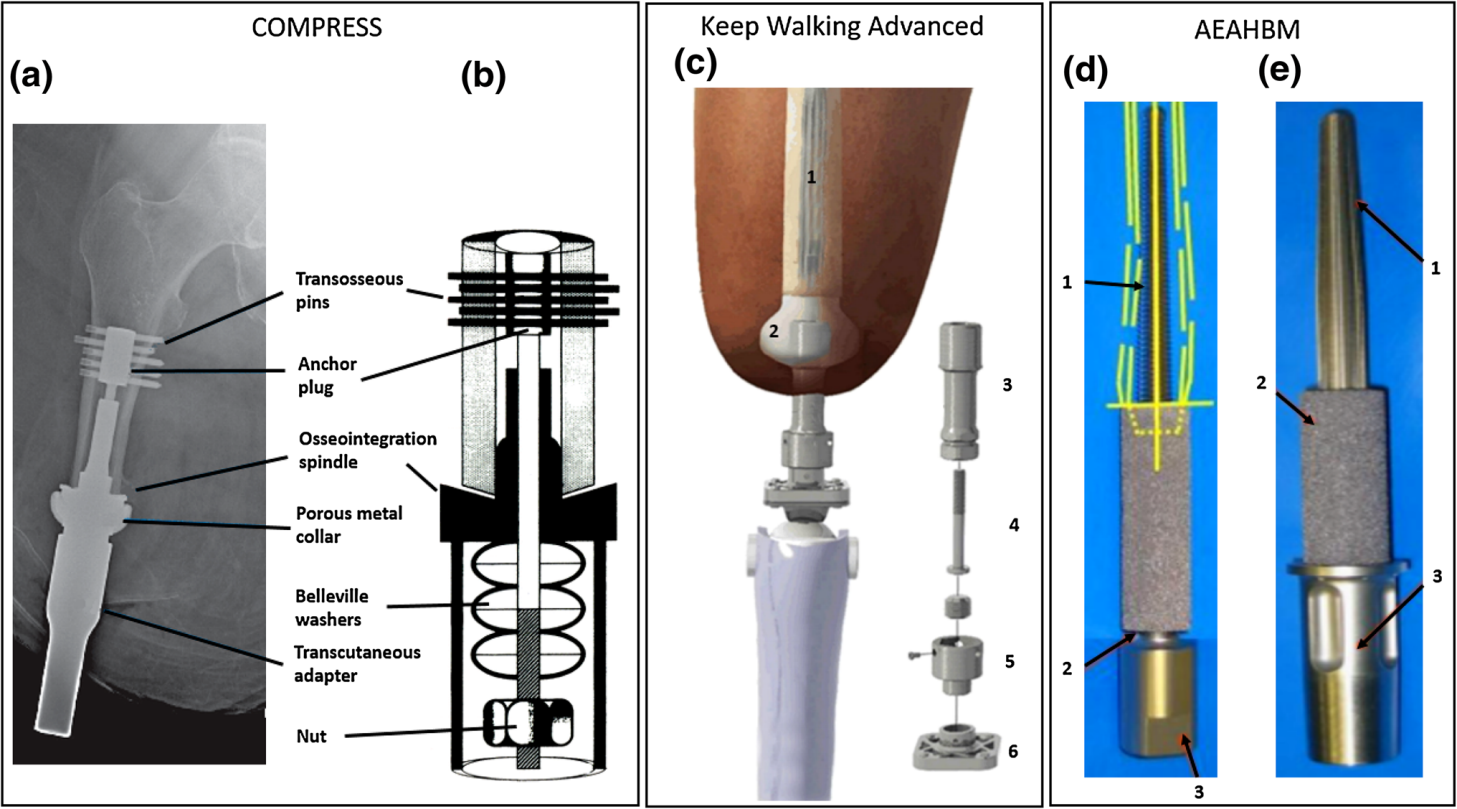
(a) Compress percutaneous device radiograph; (b) Schematic diagram of the Compress endoprosthetic implant, demonstrating Belleville washers stacked over a traction bar housed within the endoprosthetic taper at the bone-prosthetic interface; (c) Schematic view of the keep walking advanced implant system: (1) intramedullary rod, (2) spacer, (3) intermediate device, (4) locking screw, (5) upper connecting piece, (6) lower connecting piece; (d) AEAHBM implant original design; yellow lines represent the patient’s tibial shaft: (1) Tapered threaded stem of Ti6Al4 V, (2) base of porous tantalum sleeve, (3) Morse taper fitting for external prosthesis; (e) AEAHBM implant modified design: (1) Intramedullary stem of Ti6Al4 V with longitudinal splines, (2) porous tantalum sleeve, (3) external region of Ti6Al4 V implant. Image A has been adapted from Unfallchirurg^57^ with permission of Springer. Image B has been adapted from International Orthopaedics^51^ with permission of Springer. Image C is published from Rehabilitación^27^ with permission of the Publisher. Images D and E are reprinted from Veterinary Surgery^18^ with permission from John Wiley and Sons.

The eight identified implant systems are shown in Figs. 2, 3, 4, 5, and 6 and described briefly in this section. Comparisons between the systems are presented in Tables 1 and 2.

**Table 1:** Comparison of current percutaneous implant systems for direct skeletal attachment of external limb prostheses.

System	Maturity of implant system	Amputation levels	Number of surgeries	Time between S1 and S2 (months)	Total recovery time (months)
OPRA	Clinically available	Tibia, femur, humerus, radius/ulna, thumb, finger	1 or 2	3–6	3–18^a^
ILP	Clinically available	Tibia, femur, humerus	2	1.5–2	2.5–4.5^8^,^10^,^49^
OPL	Clinically available	Tibia, femur	1 or 2	1.5–2	4.5^8^
ITAP	Pre-CE mark clinical trial	Femur, humerus	1	–	–
POP	FDA early feasibility study	Femur	2	1.5^13^	–
Compress	Custom device	Femur, humerus	1 or 2	3–4^57^	–
Keep walking advanced	Clinical trial	Femur	2	3^27^	–
AEAHBM	Case study in dog	–	1	–	–

^a^12–18 months for transfemoral and transtibial amputations, 10–12 months for transhumeral amputations, 9 months for transradial amputations, and 3 months for thumb amputations

**Table 2:** Comparison of material and interface characteristics of current percutaneous implant systems for direct skeletal attachment of external limb prostheses.

System	Material	Interfaces		
		Bone -implant	Implant -percutaneous part	Percutaneous part - soft tissue
OPRA	Ti6Al4 V	Thread	Press-fit and locking screw	Polished
ILP	cobalt-chrome-molybdenum	Press-fit	Press-fit and locking screw	Polished
OPL	Ti6Al4 V	Press-fit	Press-fit and locking screw	Polished
ITAP	Ti6Al4 V	Press-fit	–	Perforated flange/polished
POP	Ti6Al4 V	Press-fit	Press-fit and locking screw	Ceramic coating
Compress	Ti6Al4 V	Transverse pins/axial compression	Taper connection	Porous titanium/polished
Keep walking advanced	Ti6Al4 V	Press-fit	Press-fit and locking screw	Polished
AEAHBM	Ti6Al4 V	Thread/press-fit	–	Porous tantalum

### OPRA

In 1998, the Swedish implant system, surgical technique, and postoperative rehabilitation protocol was standardized for transfemoral amputees by the introduction of the OPRA treatment protocol. Similar standardisation has also been done for transhumeral and thumb/finger amputations. Apart from the standardised implant versions, custom-made implants are available for the aforementioned levels, as well as for transtibial and transradial amputations. The OPRA implant system mainly consists of three components: (1) an externally threaded, cylinder-like, fully implanted component known as a “fixture”, where osseointegration takes place; (2) a percutaneous component called “abutment”, which is press-fit into the distal end of the fixture and to which the limb prosthesis is attached; and (3) an “abutment screw”, which extends through the hollow centre of the abutment to clamp the abutment and the fixture together by the proximal thread engagement to the fixture and the abutment screw head on the distal end of the abutment (Fig. 2a). Loads are transferred from the limb prosthesis to the abutment, then from the abutment to the fixture, and finally from the fixture to the bone. Implantation is normally done in two separate surgeries, but it can also be done in a single surgery on patients who have acceptable bone quality and compliance.^56^ Since its introduction, the OPRA implant system has undergone several design changes. The material has been changed from commercially pure titanium to the stronger medical grade titanium alloy Ti6Al4 V. A surface treatment of the exterior of the fixture called BioHelix™ (Fig. 2b) was added to induce a nanoporous structure for improved osseointegration.^15^ In addition to the design changes, the surgical technique for lower limb amputations was modified to implant the fixture 20 mm countersunk into the bone to address the problem of distal bone resorption, which was sometimes observed when the fixture was placed flush with the distal bone end,^60^,^72^,^75^ and to reduce the risk of infection in the bone-fixture interface.^73^ Because a fixture failure requires a major surgical intervention, the system was designed to ensure that the abutment and abutment screw fracture before the fixture, or the bone, should the system be exposed to excessive loads, as these components are more easily replaced than the fixture. Additionally, the lower limb systems and the transhumeral systems are protected by a safety device that is attached between the distal end of the abutment and the limb prosthesis. The safety device automatically releases the connection with the external prostheses if exposed to excessive loads, thus protecting the implant system from exposure to fracture inducing forces. In the studied literature, fatigue failure of fixtures has been reported in a transfemoral^64^ and a transradial patient,^63^ both using an early design of the implant system. Mechanical complications leading to revision of the abutment or the abutment screw have also been reported,^14^,^55^ but no measurement of the mechanical failure rate has been published.

The OPRA implant system has been recently enhanced at the transhumeral level to allow bidirectional communication with implanted neuromuscular interfaces for the closed-loop control of arm prosthesis.^61^ In current clinical trials, this novel osseointegrated interface has, for the first time, allowed the connection of an arm prosthesis to the patient’s bone, nerves, and muscles. This is currently the only neuroprosthetic system that enables patients to operate a prosthetic arm in daily life receiving sensory feedback* via* direct nerve stimulation.^62^

### ILP

The ILP implant system was designed for transfemoral amputees, but tibial and humeral implantation has also been reported.^10^,^11^,^40^ The implant has a cast stem of cobalt-chrome-molybdenum alloy and the implant stem is 140–180 mm long and slightly curved to prevent rotation in the intramedullary cavity and to fit to the normal curvature of the femur. Since its introduction, the system has gone through several design changes. Both the intramedullary part of the implant and the subdermal part was initially covered with a 1.5 mm thick macroporous surface known as “spongiosa metal”, consisting of tripod-like structures (Fig. 2d).^1^,^23^ The implant also had a bone-stabilising bracket, which wrapped around the cortical bone distally (Figs. 3a and 3b). As the bracket and the macroporous surface towards the soft tissue were found to cause complications, they were removed in design versions 2 and 3, respectively (Figs. 3b and 3c).^3^,^49^ The latter design was introduced in Germany 2009^49^ and subsequently in the Netherlands in 2009^53^,^77^ and in Australia in 2010.^3^ Mechanical failures of the intramedullary implant have been reported in all three countries.^5^,^23^,^49^

### OPL

The OPL was introduced in Australia in 2013^6^ and in the Netherlands in 2015.^23^ The system is used for transfemoral or transtibial amputation levels. Standardised implants are used for transfemoral amputees with sufficient stump length (≥ 160 mm), while custom-made implants are available for transtibial amputees and transfemoral amputees with very short stumps (Fig. 4).^23^ This implant system has two standard designs: OPL type A, with an extramedullary head (Fig. 4a), and OPL type B, with a flared intramedullary head for distal transfemoral amputations^23^ (Fig. 4b).

The changes from the ILP-3 to the OPL implant design include material change to Ti6Al4 V, the introduction of 1 mm high sharp longitudinal splines proximally, and change from the macroporous “spongiosa metal” surface to a plasma-sprayed rough titanium coating distally where osseointegration is desired.^2^,^4^,^6^

### POP

The POP system was developed in Salt Lake City, USA. The system is currently being evaluated in an early feasibility study in 10 transfemoral amputee subjects.^13^,^20^,^68^ It is a modular system, implanted in two separate surgeries. No further details have been published regarding the design or the surgical protocol. The human trial was preceded by animal studies of load-bearing limb prostheses in sheep.^41^,^43^–^46^,^69^ However, the implant system used in the animal trials is considerably different from the human system design. The implant system in the animal trial consists of a single component made of Ti6Al4 V (Fig. 5a). The intramedullary part is divided into three regions: a smooth region proximally, a ribbed region in the middle, and a porous coated region distally. The porous coating is combined with a collar shape to interface against the distal end of the bone.

### ITAP

The ITAP implant system is under development in the United Kingdom. A pre-CE mark clinical study for transfemoral amputees^71^ has been started, but no results have yet been published. The published results of the ITAP system include a case study from a two-year follow-up of a transhumeral amputee,^50^ and a clinical and functional outcome report from implantation of custom made implant systems in four dogs.^22^ The ITAP is a single-component system implanted in a single surgery. Similar to the other implant systems, with the exception of the ILP, the implant is made of the titanium alloy Ti6Al4 V. The proximal region of the intramedullary part of the ITAP has longitudinal cutting flutes (Fig. 5c) aimed to improve rotational stability. The subdermal and distal regions of the intramedullary part of the implant have a hydroxyapatite coating to promote soft tissue ingrowth and bone-anchorage. Since there are no publications available from the clinical study, it is unclear whether the bone anchorage is obtained with or without bone cement. Another design characteristic of the ITAP system is a subdermal porous flange towards the distal end of the residual stump to serve as a platform for soft tissue ingrowth and skin attachment to minimise the relative movement at the percutaneous interface.^22^,^50^,^66^

### COMPRESS

The COMPRESS system (Figs. 6a and 6b) was first developed as an endo-prosthetic system for oncologic limb salvage reconstruction by Biomet Corporation (now Zimmer Biomet, Warsaw, USA). The intramedullary part of the implant is attached to the bone by transverse pins in a bone-anchor plug. A porous coated collar designed to promote osseointegration is located at the distal interface of the amputated bone. To enhance osseointegration and to prevent stress-shielding of the bone, the concept of compliant pre-stress is utilised, exposing the bone-collar interface to a compressive force. Under a FDA custom device exception, a percutaneous version of this system enabling attachment of an external limb prosthesis has been developed and implanted in 10 transfemoral amputees and one transhumeral amputee. Both single-stage and two-stage surgeries have been used for implantation of the system.^57^ Two cases of periprosthetic fractures caused by falls have been reported among the transfemoral subjects.^57^

### Keep Walking Advanced

This system is under development in Valencia, Spain. It is an extension of the Keep Walking system for socket stabilization in transfemoral amputees. In the Keep Walking system, an intramedullary titanium rod is press-fit into the femur to allow for osseointegration.^28^ The distal end of the rod is connected to a large subdermal component, which serves to transfer the load from the femur and distribute it evenly to the socket of the prosthesis to avoid discomfort and soft tissue damage. In the Keep Walking Advanced system (Fig. 6c), a percutaneous extension is added to the subdermal implant in a second surgery. The extension allows for skeletal attachment of an external prosthesis, eliminating the need for the compression socket. The Keep Walking Advanced system is currently being evaluated in a clinical trial. A single case study of a 38-year-old female transfemoral amputee who received the system in 2013 has been reported,^27^ but no information about functional outcome has been presented.

### AEAHBM

The AEAHBM implant system developed in Denver, Colorado was custom-made for a single surgery implantation of a single-component system into both pelvic limbs of a dog^18^ (Fig. 6d). One of the implants had to be removed because of failed osseointegration after 14 months, but a redesigned implant (Fig. 6e) was implanted with successful results up to the time of the report, 26 months after the initial surgery. The original implant consisted of a threaded tapered intramedullary stem consisting of Ti6Al4 V. The distal end of the stem was covered by a porous tantalum sleeve to allow for soft tissue integration while also serving as a collar towards the distal end of the bone. In the redesigned system, the tapered thread was exchanged for an unthreaded stem with longitudinal splines in order to provide more rotational stability.

### Number of Treated Human Subjects

There is only limited information in peer-reviewed publications regarding the number of patients who have been recipients of implant systems for bone-anchored limb prostheses. It has been reported that approximately 150 patients were treated with the OPRA system in Sweden between 1990 and 2009^34^; approximately 150 patients had been treated with the ILP system in Germany, the Netherlands, and Australia until 2016^3^; and at least 22 patients were treated with the OPL system in Australia between November 2013 and December 2014.^6^ Since those reports, more patients have been treated with each of these systems. Oral and unverified online reports indicate that the current number of patients treated with each of the clinically available systems is in the hundreds; however, the actual numbers must be verified in formally reported clinical studies. For the other systems, the number of treated patients are lower. The peer-reviewed literature has only reported a single subject treated with the ITAP system,^50^ 11 subjects treated with the COMPRESS system,^57^ and a single subject treated with the keep walking advanced^27^ system. According to oral and unverified online reports, a further 20 transfemoral patients have been treated with the ITAP system in the clinical trial, and at least eight people have been treated with the POP system in the early feasibility study.

### Surgical Approach

Of the systems implanted in human subjects, ITAP is the only system that is always implanted in a single surgery. The other systems have mostly followed two-stage surgical protocols, although single-stage procedures have been reported for the OPRA, the OPL and the COMPRESS systems.^7^,^56^,^57^ In the first stage, an incision is made in the distal end of the stump, the residual bone is reamed and prepared for insertion of the implant, and the implant is then inserted and the skin is closed. The bone and the skin are allowed to heal for a period of time to enable osseointegration between the bone and the implant. In the second stage, the percutaneous part is inserted with its proximal end into the implanted component and the distal end extending through the skin.

### Rehabilitation

Different healing and rehabilitation times are used before the implant can be fully loaded. A schedule is followed in which progressively higher loads are applied to the external part of the implant system until full loading with the external prosthesis is eventually allowed.^8^,^30^,^48^,^50^ It is important to have a close collaboration between the physiotherapist and prosthetist to monitor the rehabilitation of the patient and to ensure that the prosthetic components are carefully selected and aligned. It is recommended that a prosthetic knee component providing effortless flexion and controlled extension is used before full weight-bearing is allowed.^30^

The recommended period between the first surgery and the time when the patient is able/allowed to fully load the system with an external prosthesis varies among individuals, implant systems, and amputation level. According to the standard OPRA protocol, this period should be approximately 12 months for transfemoral amputees,^30^,^56^ while for the ILP and the OPL, full weight bearing on the prosthesis is recommended after 2.5–3 and 4–5 months, respectively.^8^,^10^,^49^ For the other systems, the number of cases reported in this regard is too small.

### Bone-implant Interface

In order to have a long-term successful outcome, it is essential to obtain a stable connection between the implant and the bone. It is believed that small relative movements between the implant and the bone can cause the formation of a fibrous layer around the implant, leading to mechanical instability and the need for implant revision. Different approaches have been used to achieve a stable connection between the implant and the bone. Osseointegration is often cited as the underlying working mechanism, but limited evidence has been provided in this regard. Verification of achieved osseointegration would preferably include X-ray analysis and radiostereometric analysis (RSA),^60^ as well as high-resolution interface analysis after implant retrieval.^64^ The only system that has provided such evidence in peer-reviewed literature is the OPRA implant system.^60^,^64^

Three different anchoring strategies were found in the studied systems: (1) a threaded connection, which is utilised in the OPRA and the original AEAHBM system; (2) a press-fit interface, which is present in the ILP, OPL, ITAP, POP, Keep Walking Advanced, and the redesigned AEAHBM system; and (3) a bone anchor with transverse intraosseous pins in combination with a compressive force and bone-ingrowth at the bone-collar interface, as is used in the COMPRESS system. A threaded connection inherently has good mechanical stability in the longitudinal direction. Initial rotational stability is achieved by friction and long-term stability is achieved by a combination of friction and mechanical interlocking, where the bone tissue grows into macro, micro, and nano “irregularities” on the implant surface. The press-fit interface has a lower longitudinal axial stability, initially relying solely on friction and in the long term relying on both friction and bone ingrowth for longitudinal and rotational stability. A bone anchor with transverse intraosseous pins, in combination with a compressive bone collar with bone ingrowth promoting surface properties, naturally creates a high mechanical stability, both in the longitudinal and the rotational directions. In order to create a more stable interface between the implant and the bone, additional features have been added to some of these systems. These include longitudinal splines or fluted regions for improved rotational stability as in OPRA, OPL, ITAP, POP, Keep Walking Advanced and the AEAHBM systems; a curvature along the longitudinal axis, as is utilized in the ILP and the OPL systems; and a flared, or collared interface at the distal bone interface, as used in all systems except OPRA. Geometrical fit between the implant and the bone appears to be crucial for a successful outcome as observed by loosening and failure of undersized implants,^5^,^8^ or fracture at the insertion of oversized implants.^44^ Bone ingrowth is dependent on the implant material, the implant design, and the geometrical fit in the bone.^9^ In all of the studied implant systems except the ILP, the bulk material of the implant is the titanium alloy Ti6Al4 V. In the ILP, a cobalt-chrome-molybdenum alloy is used instead. Porous exterior surfaces are used locally in all implant systems to enhance the bone ingrowth. The OPRA system has the laser-induced nanoporous microstructure BioHelix™ surface, the ILP system has a macroporous spongiosa surface, and the OPL has a rough surface of plasma sprayed titanium on the distal half of the implant. Similarly, the distal region of the POP implant, including a collar at the interface toward the distal end of the bone, is covered by a porous layer of pure titanium. The COMPRESS system utilises a hydroxyapatite porous collar in combination with a compressive force of 1.8-3.6 kN^57^,^65^ across the interface, while the AEAHBM has a collar of porous tantalum toward the distal bone end. In the Keep Walking Advanced system, small holes are located on the proximal end of the UHMWPE (ultra-high-molecular-weight polyethylene) spacer at the interface toward the distal end of the bone.^26^ The ITAP has a coating of hydroxyapatite on the distal part of the intramedullary portion of the implant.

### Implant-percutaneous Part Interface

In the OPRA system, the abutment is connected to the fixture by a smooth surface press-fit. The abutment is also clamped to the fixture by a preload from the abutment screw with a thread engagement with the fixture proximal to the abutment.

The ILP and OPL systems have a press-fit Morse taper connection between the implant and the percutaneous part, called the dual-cone adapter. A safety screw inserted longitudinally from the dual-cone adapter to the implant provides additional locking of the components.

In the POP system used in the ongoing human trial, the implant is connected to the exterior of the body by a ceramic-coated percutaneous post, which is clamped to the implant with a locking screw.^12^

The porous coated collar in the COMPRESS system is connected to the bone anchor by a traction rod and a compression nut, which, in combination with a number of Belleville washers, apply a compressive force at the distal bone end.

In the Keep Walking Advanced system, the implant is attached to the percutaneous extension by a tapered interface and a locking screw, which has a proximal thread engagement with the implant.^26^

### Percutaneous Interface

The percutaneous component of the OPRA system is the abutment, which has a smooth polished surface to minimise contact and friction at the skin interface. In the ILP and the OPL systems, the percutaneous interface consists of the dual-cone adapter and, in some designs, also the distal end of the implant. The percutaneous surfaces of the implant are smooth-polished and have a niobium-oxide coating aimed to minimise soft tissue adhesion at the percutaneous interface.^5^,^23^

In the ITAP system, the percutaneous interface is stabilised by the subdermal porous flange, allowing for soft tissue ingrowth and suture of the thinned skin flap to minimise relative movement.^50^ Distal to the porous flange, the peg for attachment of the external prosthesis is coated with a low-friction DLC (diamond-like carbon) surface coating to reduce bacterial adhesion.^22^,^50^,^66^

The COMPRESS and the AEAHBM systems both have a porous subdermal surface distal to the bone to promote soft tissue integration and to provide a soft tissue seal to the implant. In the COMPRESS system, the porous coating consists of titanium, while the AEAHBM system uses tantalum.^13^,^18^,^57^ The percutaneous interface of the POP and the COMPRESS systems are characterised by smooth low-friction surfaces, similar to the other implant systems for human implantation.

### Safety Devices

Safety precautions have been taken to protect the bone and the implant from direct exposure to high loads, especially in the event of a fall. The OPRA implant system has different safety devices for different amputation levels. They are separate components, which are connected between the abutment and the external prosthesis and automatically release the connection between the implant and the prosthesis if they are exposed to a load exceeding a pre-set limit. All OPRA safety systems protect against excessive torque around the longitudinal axis, while the transfemoral system also protects from excessive bending moments in the normal plane of the longitudinal axis. Release limits for the systems are set to be high enough to avoid release during normal use, but low enough to ensure that the bone or the implant system is not damaged. The limits are set to 15 Nm torque and 70 Nm bending moment. In case of release, the safety system can be restored to normal operation by the patient without the need to meet a prosthetist. The ILP and OPL safety systems are protecting from high torques being transferred to the implant by a connection adapter^25^ or a click safety adapter^23^,^77^ attached between the distal end of the dual-cone adapter and the external prosthesis. The connection adapter is equipped with a safety mechanism, consisting of one or several shear pins, designed to break in case the implant is exposed to high torsional loads.^11^ If this happens, replacement of the broken part can be done in a clinical setting.^3^ There is limited information about the safety systems for the other implant systems. However, the case report about the transhumeral ITAP patient mentions that the ITAP is equipped with a safety component, mounted between the implant and the prosthesis, which is designed to break at loads corresponding to 10 kg or more.^50^

## Discussion

Several systems have been developed for bone-anchored limb prostheses and different approaches have been employed to achieve a stable attachment between the implant and the bone. Comparing the ILP and OPL implant systems with the OPRA implant system reveals that the latter follows a slower rehabilitation protocol for lower limb amputations. This could indicate that a longer time is needed to achieve a mechanically stable interface for a threaded implant than for a press-fit implant. However, the historical development of the implants must also be considered. The threaded implant design used in the OPRA system was developed using experience gained from the dental industry as the first and longest user of osseointegration, whereas the press-fit implant design originates from intramedullary hip implants, which have a tradition of fixation to the bone by press-fit in combination with bone cement.

The OPRA system follows a conservative approach, with a healing period before any load is applied to the implant. This is based on experimental results from animal studies, which have shown that the mechanical capacity of the bone-implant interface for threaded implants is improved by healing under unloaded conditions in both torsion and pull-out load.^17^,^47^ For the pioneers in the field of bone-anchored limb prostheses, the highest priority during the development has been to achieve a successful end result, rather than rehabilitation speed. The primary stability is determined by the geometrical fit between the implant and the bone, the bone quality and quantity, and thus varies between individuals. If the primary stability of the implant after the surgery is sufficient to withstand initial loading, the rehabilitation could start earlier and significantly reduce the total recovery time. On the other hand, if the primary stability is insufficient, the reduced weakness of the bone-implant interface during the initial healing would potentially lead to a higher incidence of early fixture loosening if such a reduced healing time protocol was adopted.

Another difference between the clinically available implant systems is the intramedullary length of the implant (140–180 mm for ILP, 160 mm for OPL, and 80 mm for OPRA). This indicates that press-fit implants require a longer intramedullary length to achieve a stable connection in the longitudinal direction than a threaded implant. A shorter implant length is advantageous, as it imposes fewer eligibility constraints on the residual stump length. Furthermore, in the event of a catastrophic failure, with the worst-case scenario requiring re-amputation above the implant, a shorter intramedullary length leads to a longer residual length of the stump after the re-amputation surgery.

To achieve bone ingrowth, the trend is to use the titanium alloy Ti6Al4 V as the bulk material in combination with porous surface on the implant. The degree of porosity varies between systems, but inevitably comes with a trade-off by having reduced mechanical strength and fatigue properties in these regions. Most of the systems used in humans are modular, which limits the mechanical capacity but simplifies the surgical intervention in case a revision surgery is needed. To avoid irritation and superficial infections, which could potentially lead to more severe deep infections, two approaches are employed at the percutaneous interface in the different implant systems. The first approach is to try to prevent skin and soft tissue adhesion by having a polished surface on the percutaneous component as in the OPRA, OPL, POP, Keep Walking Advanced and later designs of the ILP system. In the OPRA system, this approach is combined with suture of the skin directly to the distal end of the bone at the percutaneous interface in order to minimize relative movement and to create an infection barrier. The other approach is to create an infection barrier by promoting soft-tissue ingrowth to the percutaneous component. This approach is used in the ITAP, COMPRESS, AEAHBM, and earlier designs of the ILP systems, by having a porous surface on the percutaneous component.

The scientific literature contains limited information about the mechanical features of bone-anchoring implant systems. Reporting the performance of such systems under mechanical stress in bench tests, or activities of the daily living, has been given a low priority by the development teams in favor of clinical outcomes. The available literature refers to osseointegration as the responsible mechanism that allows direct skeletal attachment of limb prostheses; however, insufficient evidence has been provided for most of the implant systems on the degree of osseointegration achieved with the different designs.

It is difficult to compare the available systems because they have undergone several changes over time, and clinical trials continue to be limited. The three design principles known at present are fundamentally different and pose particular advantages and disadvantages, which might in the future serve as the basis to determine which approach is most suitable for particular subjects.

## Conclusion

Current systems for bone anchoring of limp prostheses use intramedullary implants. Primary stability between bone and implant is achieved by one of three strategies: a threaded connection, a press-fit connection, or axial compression. Secondary stability is achieved by bone ingrowth into porous surfaces of the implant. Although there are large differences between current implant systems, the three clinically available systems (OPRA, ILP, and OPL) have shown functional improvements for patients with socket-related issues. Recent developments of implant systems, surgical protocols, and safety devices have reduced the rate of mechanical failure and infectious complications. Moreover, further improvements are likely to continue based on field data and information from the ongoing human trials. Future developments are likely to address several factors, such as the perceived long rehabilitation time before loading, the need for two separate surgeries, the incidence of superficial infection at the percutaneous interface, mechanical failures in highly demanding activities, and the possibility of additionally providing closed-loop, neuro-muscular control of the limb prostheses.

## Abbreviations

AEAHBM: Alameda East Animal Hospital BioMedtrix
ILP: Integral leg prosthesis
ITAP: Intraosseous transcutaneous amputation prosthesis
OPL: Osseointegrated prosthetic limb
OPRA: Osseointegration prostheses for the rehabilitation of amputees
POP: Percutaneous osseointegrated prosthesis
UHMWPE: Ultra-high-molecular-weight polyethylene

## References

[1] Ahlers, O. Verfahren zur herstellung eines implantates mit einer seine oberfläche zumindest teilweise bedeckenden metallischen offenzelligen struktur. Patent: EP 0,502,349, 1995.

[2] Al Muderis, M. An osseointegrable device. Patent: US patent app. publ. 0,331,422, 2016, 2016.

[3] Al Muderis M, Aschoff HH, Bosley B, Raz G, Gerdesmeyer L, Burkett B (2016). Direct skeletal attachment prosthesis for the amputee athlete: The unknown potential. Sport. Eng..

[4] Al Muderis M, Bosley BA, Florschutz AV, Lunseth PA, Tyler DK, Highsmith JM, Kahle JT (2016). Radiographic assessment of extremity osseointegration for the amputee. Technol. Innov..

[5] Al Muderis M, Khemka A, Lord SJ, Van de Meent H, Frölke JPM (2016). Safety of osseointegrated implant for transfemoral amputees, a two-center prospective cohort study. J. Bone Jt. Surg..

[6] Al Muderis M, Lu W, Li JJ (2017). Osseointegrated Prosthetic Limb for the treatment of lower limb amputations : Experience and outcomes. Unfallchirurg.

[7] Al Muderis M, Lu W, Tetsworth K, Bosley B, Li JJ (2017). Single-stage osseointegrated reconstruction and rehabilitation of lower limb amputees: The Osseointegration Group of Australia Accelerated Protocol-2 (OGAAP-2) for a prospective cohort study. BMJ Open.

[8] Al Muderis M, Tetsworth K, Khemka A, Wilmot S, Bosley B, Lord SJ, Glatt V (2016). The Osseointegration Group of Australia Accelerated Protocol (OGAAP-1) for two-stage osseointegrated reconstruction of amputated limbs. Bone Joint J..

[9] Albrektsson T, Brånemark P-I, Hansson H-A, Lindström J (1981). Osseointegrated titanium implants: requirements for ensuring a long-lasting, direct bone-to-implant anchorage in man. Acta Orthop. Scand..

[10] Aschoff HH (2010). Transcutaneous, distal femoral, intramedullary attachment for above-the-knee prostheses: an endo-exo device. J. Bone Jt. Surg..

[11] Aschoff H (2017). TOPS—transkutane osseointegrierte Prothesensysteme. Orthopädie und Unfallchirurgie up2date.

[12] Bachus, K., S. Jeyapalina, J. P. Beck, R. Bloebaum, J. P. Agarwal, J. A. Longo, E. Kubiak, and B. Mueller Holt. Percutaneous osseointegrated implant assembly for use in supporting an exo-prosthesis. Patent: US 9,433,505, 2016.

[13] Beck, J., S. Sinclair, B. Gillespie, B. Darter, J. Agarwal, P. Stevens, S. Turley, and E. Kubiak. Early observations of a Federal Drug Administration feasibility study determining the safety and efficacy of a percutaneous osseointegrated prosthesis system. 7th international conference: advances in orthopaedic osseointegration, San Diego, 2017.

[14] Brånemark R, Berlin Ö, Hagberg K, Bergh P, Gunterberg B, Rydevik B (2014). A novel osseointegrated percutaneous prosthetic system for the treatment of patients with transfemoral amputation: a prospective study of 51 patients. Bone Jt. J..

[15] Brånemark R, Emanuelsson L, Palmquist A, Thomsen P (2011). Bone response to laser-induced micro- and nano-size titanium surface features. Nanomed. Nanotechnol. Biol. Med..

[16] Brånemark PI, Hansson BO, Adell R, Breine U, Lindström J, Hallén O, Öhman A (1977). Osseointegrated implants in the treatment of the edentulous jaw. Experience from a 10-year period. Scand. J. Plast. Reconstr. Surg. Suppl..

[17] Brånemark R, Öhrnell LO, Nilsson P, Thomsen P (1997). Biomechanical characterization of osseointegration during healing: An experimental *in vivo* study in the rat. Biomaterials.

[18] Drygas KA, Taylor R, Sidebotham CG, Hugate RR, McAlexander H (2008). Transcutaneous tibial implants: A surgical procedure for restoring ambulation after amputation of the distal aspect of the tibia in a dog. Vet. Surg..

[19] Dudek NL, Marks MB, Marshall SC, Chardon JP (2005). Dermatologic conditions associated with use of a lower-extremity prosthesis. Arch. Phys. Med. Rehabil..

[20] Encore Medical, L. P. Early Feasibility Study of the Percutaneous Osseointegrated Prosthesis (POP)—Full Text View—ClinicalTrials.gov, 2016, at http://clinicaltrials.gov/ct2/show/study/NCT02720159

[21] Eriksson E, Brånemark P-I (1994). Osseointegration from the perspective of the plastic surgeon. Plast. Reconstr. Surg..

[22] Fitzpatrick N, Smith TJ, Pendegrass CJ, Yeadon R, Ring M, Goodship AE, Blunn GW (2011). Intraosseous transcutaneous amputation prosthesis (ITAP) for limb salvage in 4 dogs. Vet. Surg..

[23] Frölke JPM, Leijendekkers RA, van de Meent H (2017). Osseointegrated prosthesis for patients with an amputation. Unfallchirurg.

[24] Frossard L, Hagberg K, Häggström E, Gow DL, Brånemark R, Pearcy M (2010). Functional outcome of transfemoral amputees fitted with an osseointegrated fixation: Temporal gait characteristics. JPO J. Prosthetics Orthot..

[25] Grundei, H. Connection adapter. Patent: US 8,226,731, 2012.

[26] Guirao, L. Modular femoral endoprosthesis. Patent: EP Patent application 2,138,133, 2009.

[27] Guirao L, Samitier B, Alos J, Tibau R, Pleguezuelos E (2017). Osteointegración con el sistema keep walking advanced. Rehabilitación.

[28] Guirao L, Samitier CB, Costea M, Camos JM, Majo M, Pleguezuelos E (2016). Improvement in walking abilities in transfemoral amputees with a distal weight bearing implant. Prosthet. Orthot. Int..

[29] Hagberg K, Brånemark R (2001). Consequences of non-vascular trans-femoral amputation: A survey of quality of life, prosthetic use and problems. Prosthet. Orthot. Int..

[30] Hagberg K, Brånemark R (2009). One hundred patients treated with osseointegrated transfemoral amputation prostheses -Rehabilitation perspective. J. Rehabil. Res. Dev..

[31] Hagberg K, Häggström E, Uden M, Brånemark R (2005). Socket versus bone-anchored trans-femoral prostheses: Hip range of motion and sitting comfort. Prosthet. Orthot. Int..

[32] Hagberg K, Hansson E, Brånemark R (2014). Outcome of percutaneous osseointegrated prostheses for patients with unilateral transfemoral amputation at two-year follow-up. Arch. Phys. Med. Rehabil..

[33] Häggström E, Hagberg K, Rydevik B, Brånemark R (2013). Vibrotactile evaluation: osseointegrated versus socket-suspended transfemoral prostheses. J. Rehabil. Res. Dev..

[34] Häggstrom E, Hansson E, Hagberg K (2013). Comparison of prosthetic costs and service between osseointegrated and conventional suspended transfemoral prostheses. Prosthet. Orthot. Int..

[35] Hall CW (1974). Developing a permanently attached artificial limb. Bull. Prosthet. Res..

[36] Hall C (1976). Skeletal extension development: criteria for future designs. Bull. Prosthet. Res..

[37] Hall CW (1985). A future prosthetic limb device. J. Rehabil. Res. Dev..

[38] Hall CW, Mallow A, Cox PA (1977). Developing a supraperiostial endoprosthesis. Trans. Am. Soc. Artif. Intern. Organs.

[39] Hillock R, Keggi J, Kennon R, McPherson E, Terry C, Brasil D, McTighe T (2013). A global collaboration—osteointegration implant (OI) for transfemoral amputation. JISRF Reconstr. Rev..

[40] Hoffmeister T, Schwarze F, Aschoff HH (2017). Das Endo-Exo-Prothesen versorgungskonzept: verbesserung der lebensqualität nach extremitätenamputation. Unfallchirurg.

[41] Holt BM, Bachus KN, Beck JP, Bloebaum RD, Jeyapalina S (2013). Immediate post-implantation skin immobilization decreases skin regression around percutaneous osseointegrated prosthetic implant systems. J. Biomed. Mater. Res. Part A.

[42] Jacobs R, Brånemark R, Olmarker K, Rydevik B, Van Steenberghe D, Brånemark PI (2000). Evaluation of the psychophysical detection threshold level for vibrotactile and pressure stimulation of prosthetic limbs using bone anchorage or soft tissue support. Prosthet. Orthot. Int..

[43] Jeyapalina S, Beck JP, Bachus KN, Bloebaum RD (2012). Cortical bone response to the presence of load-bearing percutaneous osseointegrated prostheses. Anat. Rec..

[44] Jeyapalina S, Beck JP, Bachus KN, Chalayon O, Bloebaum RD (2014). Radiographic evaluation of bone adaptation adjacent to percutaneous osseointegrated prostheses in a sheep model. Clin. Orthop. Relat. Res..

[45] Jeyapalina S, Beck JP, Bachus KN, Williams DL, Bloebaum RD (2012). Efficacy of a porous-structured titanium subdermal barrier for preventing infection in percutaneous osseointegrated prostheses. J. Orthop. Res..

[46] Jeyapalina S, Beck JP, Bloebaum RD, Bachus KN (2014). Progression of bone ingrowth and attachment strength for stability of percutaneous osseointegrated prostheses. Clin. Orthop. Relat. Res..

[47] Johansson CB, Albrektsson T (1987). Integration of screw implants in the rabbit: A 1-year follow-up of removal torque of titanium implants. Int. J. Oral Maxillofac. Implant..

[48] Jönsson S, Caine-Winterberger K, Brånemark R (2011). Osseointegration amputation prostheses on the upper limbs: methods, prosthetics and rehabilitation. Prosthet Orthot Int.

[49] Juhnke D-L, Beck JP, Jeyapalina S, Aschoff HH (2015). Fifteen years of experience with integral-leg-prosthesis: Cohort study of artificial limb attachment system. J. Rehabil. Res. Dev..

[50] Kang NV, Pendegrass C, Marks L, Blunn G (2010). Osseocutaneous integration of an intraosseous transcutaneous amputation prosthesis implant used for reconstruction of a transhumeral amputee: Case report. J. Hand Surg. Am..

[51] Kramer MJ, Tanner BJ, Horvai AE, O’Donnell RJ (2008). Compressive osseointegration promotes viable bone at the endoprosthetic interface: Retrieval study of Compress^®^ implants. Int. Orthop..

[52] Legro MW, Reiber G, del Aguila M, Ajax MJ, Boone DA, Larsen JA, Smith DG, Sangeorzan B, Aguila M, Megan J, A Boone DA, Larsen JA, Smith DG (1999). Issues of importance reported by persons with lower limb amputations and prostheses. J. Rehabil. Res. Dev..

[53] Leijendekkers RA, Staal JB, van Hinte G, Frölke JP, van de Meent H, Atsma F, Nijhuis-van der Sanden MWG, Hoogeboom TJ (2016). Long-term outcomes following lower extremity press-fit bone-anchored prosthesis surgery: a 5-year longitudinal study protocol. BMC Musculoskelet. Disord..

[54] Leijendekkers RA, van Hinte G, Frölke JP, van de Meent H, Nijhuis-van der Sanden MWG, Staal JB (2017). Comparison of bone-anchored prostheses and socket prostheses for patients with a lower extremity amputation: A systematic review. Disabil. Rehabil..

[55] Lennerås M, Tsikandylakis G, Trobos M, Omar O, Vazirisani F, Palmquist A, Berlin Ö, Brånemark R, Thomsen P (2016). The clinical, radiological, microbiological, and molecular profile of the skin-penetration site of transfemoral amputees treated with bone-anchored prostheses. J. Biomed. Mater. Res. Part A.

[56] Li Y, Brånemark R (2017). Osseointegrated prostheses for rehabilitation following amputation: The pioneering Swedish model. Unfallchirurg.

[57] McGough RL, Goodman MA, Randall RL, Forsberg JA, Potter BK, Lindsey B (2017). The Compress^®^ transcutaneous implant for rehabilitation following limb amputation. Unfallchirurg.

[58] Meulenbelt HE, Dijkstra PU, Jonkman MF, Geertzen JH (2006). Skin problems of the stump in lower limb amputees. Disabil. Rehabil..

[59] Mooney V, Schwartz SA, Roth AM, Gorniowsky MJ (1977). Percutaneous implant devices. Ann. Biomed. Eng..

[60] Nebergall A, Bragdon C, Antonellis A, Kärrholm J, Brånemark R, Malchau H (2012). Stable fixation of an osseointegated implant system for above-the-knee amputees: titel RSA and radiographic evaluation of migration and bone remodeling in 55 cases. Acta Orthop..

[61] Ortiz-Catalan M, Håkansson B, Brånemark R (2014). An osseointegrated human-machine gateway for long-term sensory feedback and motor control of artificial limbs. Sci. Transl. Med..

[62] Ortiz-Catalan, M., E. Mastinu, R. Brånemark, and B. Håkansson. Direct neural sensory feedback and control via osseointegration. XVI World Congress of the International Society for Prosthetics and Orthotics (ISPO)., Cape Town, South Africa., 2017.

[63] Palmquist A, Jarmar T, Emanuelsson L, Brånemark R, Engqvist H, Thomsen P (2008). Forearm bone-anchored amputation prosthesis: a case study on the osseointegration. Acta Orthop..

[64] Palmquist A, Windahl SH, Norlindh B, Brånemark R, Thomsen P (2014). Retrieved bone-anchored percutaneous amputation prosthesis showing maintained osseointegration after 11 years-a case report. Acta Orthop..

[65] Pedtke AC, Wustrack RL, Fang AS, Grimer RJ, O’Donnell RJ (2012). Aseptic failure: How does the compress implant compare to cemented stems?. Clin. Orthop. Relat. Res..

[66] Pendegrass CJ, Goodship AE, Price JS, Blunn GW (2006). Nature’s answer to breaching the skin barrier: an innovative development for amputees. J. Anat..

[67] Pitkin M (2013). Design features of implants for direct skeletal attachment of limb prostheses. J. Biomed. Mater. Res. Part A.

[68] Potter BK (2016). From bench to bedside: A perfect fit? Osseointegration can improve function for patients with amputations. Clin. Orthop. Relat. Res..

[69] Shelton TJ, Peter Beck J, Bloebaum RD, Bachus KN (2011). Percutaneous osseointegrated prostheses for amputees: Limb compensation in a 12-month ovine model. J. Biomech..

[70] Sherman R (1999). Utilization of prostheses among US veterans with traumatic amputation: A pilot survey. J. Rehabil. Res. Dev..

[71] Stanmore Implants Ltd. Intraosseous transcutaneous amputation prosthesis—ClinicalTrials.gov, 2016, at https://clinicaltrials.gov/ct2/show/NCT02491424?term=ITAP&rank=1

[72] Stenlund P, Trobos M, Lausmaa J, Brånemark R, Thomsen P, Palmquist A (2016). The effect of load on the bone-anchored amputation prostheses. J. Orthop. Res..

[73] Tillander J, Hagberg K, Hagberg L, Brånemark R (2010). Osseointegrated titanium implants for limb prostheses attachments: Infectious complications. Clin. Orthop. Relat. Res..

[74] Tranberg R, Zügner R, Kärrholm J (2011). Improvements in hip- and pelvic motion for patients with osseointegrated trans-femoral prostheses. Gait Posture.

[75] Tsikandylakis G, Berlin Ö, Brånemark R (2014). Implant survival, adverse events, and bone remodeling of osseointegrated percutaneous implants for transhumeral amputees. Clin. Orthop. Relat. Res..

[76] Van de Meent H, Hopman MT, Frölke JP (2013). Walking ability and quality of life in subjects with transfemoral amputation: a comparison of osseointegration with socket prostheses. Arch. Phys. Med. Rehabil..

[77] Van de Meent H, Hopman MT, Frölke JP (2013). Walking ability and quality of life in subjects with transfemoral amputation: a comparison of osseointegration with socket prostheses. Arch. Phys. Med. Rehabil..

[78] Van Eck CF, Mcgough RL (2015). Clinical outcome of osseointegrated prostheses for lower extremity amputations: A systematic review of the literature. Curr. Orthop. Pract..

